# Factors shaping socio-emotional trajectories in sanctuary-living bonobos: a longitudinal approach

**DOI:** 10.1098/rsos.240435

**Published:** 2024-12-18

**Authors:** Stephanie Kordon, Christine E. Webb, Jake S. Brooker, Frans B.M. de Waal, Zanna Clay

**Affiliations:** ^1^ Department of Psychology, Durham University, South Road, Durham DH1 3LE, UK; ^2^ Department of Human Evolutionary Biology, Harvard University, Divinity Avenue, Cambridge, MA 02138, USA; ^3^ Department of Psychology, Emory University, Atlanta, GA 30322, USA

**Keywords:** social development, maternal loss, sociability, consolation, rearing, rehabilitation

## Abstract

Early maternal loss can have lasting detrimental effects on primate social development. While many rehabilitation settings provide enriching environments to buffer against such effects in orphans, previous research indicates that young bonobo (*Pan paniscus*) orphans exhibit striking deficiencies in socio-emotional competence compared to their mother-reared peers. However, such studies are generally cross-sectional, without accounting for changes across the lifespan. We conducted longitudinal observations in bonobos living in an accredited African ape sanctuary to examine how rearing background, sex and age predict social tendencies including affiliation, consolation and aggression risk. Affiliative tendencies increased in females and decreased in males with age but were overall lower in orphans compared to mother-reared bonobos. Consolation tendencies decreased with age in mother-reared bonobos, while orphans showed consistently lower consolation (akin to levels of older mother-reared individuals). Young and male bonobos were more likely to receive aggression, while mother-reared and older females were more likely aggressors. Our study highlights the potential that ape sanctuaries like this can have by demonstrating that orphans exhibit decreased affiliative tendencies yet show social functioning ranging within patterns of their mother-reared peers. We discuss these results in the context of bonobos’ natural social ecology and ongoing rehabilitation efforts in this species.

## 1. Introduction

As with other mammals, early maternal loss in primates can have both immediate as well as lasting detrimental effects that persist into adulthood. Longitudinal studies on wild primate populations—including baboons (*Papio cynocephalus*) in Amboseli, Kenya [1] and chimpanzees (*Pan troglodytes*) in Mahale and Gombe, Tanzania [2,3]—reveal that losing a mother early in life can lead to a variety of negative fitness consequences (e.g. survival and longevity). Moreover, the nature and severity of these fitness consequences vary according to the sex of the infant and the age when they lost their mother. For instance, compared to females, chimpanzee male survival is more affected by maternal loss after the age of weaning and into adolescence. This may reflect the paramount importance of maternal support for chimpanzee males, a philopatric species, who continue to benefit from their mother’s ecological knowledge (e.g. on resource distribution) into adulthood, while females become more independent upon preparing for dispersal [2,3]. Moreover, male chimpanzees orphaned at an immature age after weaning subsequently reproduce later and less successfully than non-orphaned males [4].

A close cousin of the chimpanzee, the bonobo (*Pan paniscus*) is another male philopatric species where maternal bonds appear to play an especially important role in offering sons life-long maternal support [5]. Through building strong and enduring coalitions with their mothers, sons can acquire and maintain high-rank positions [6], while also themselves benefitting through kin selection as well as enhanced coalitionary strength. The high social status of bonobo females and the characteristic cooperative and tolerant structure among female group members facilitate maternal support of philopatric sons [7], which in turn increases the fitness outcomes of both [5].

In addition to promoting offspring survival and reproductive success, research in humans and other animals shows that maternal support facilitates healthy social and emotional functioning. Research on neglected human children from orphanages and early foster homes suggests that maternal deprivation can negatively affect social development, including behavioural and emotional regulation (see [8]). While adoption into a family environment often helps mitigate these effects, prolonged social deprivation beyond the first six months of life prior to adoption often leads to persistent cognitive, socio-emotional and behavioural challenges into early adolescence [9–12]. However, such outcomes are not necessarily inevitable or uniform across all individuals. Recovery is also possible, especially when deprivation does not extend beyond the first six months of life [11]; moreover, various individual and environmental factors, such as genetic predispositions and the quality of subsequent caring environments, also serve as both protective and risk factors.

Although less studied, early life maternal deprivation and subsequent adverse social environments can also negatively affect the development of social competence (i.e. adequate social functioning within a social group) in other primates. Likewise, however, such effects may be buffered by social support along with other factors (e.g. [13]; see below). Studies on ex-laboratory chimpanzees, who suffered early maternal loss along with long-term deprivation in solitary housing for over 20 years, showed long-term deficits in grooming and social closeness to their peers into adulthood [14]. Captive chimpanzees who were maternally deprived in infancy (1−2 years) and socially isolated for longer were more fearful and less socially active, with higher stress responses during rehabilitation as compared to later deprived individuals (3−4 years) who were peer-reared for one year pre-laboratory isolation [15]. In former pet and entertainment chimpanzees in a primate rescue and rehabilitation centre in Spain, wild-caught individuals who additionally experienced early life adversities prior to their captive lives (such as the transfer from wild to species-inappropriate isolated captive life and potential maltreatment and malnutrition) were socially impaired, including reduced affiliative behaviour and grooming activity compared to their captive-born peers [16,17]. The play behaviour of orphaned juvenile chimpanzees (rescued victims of the illegal pet and bushmeat trades) in a naturalistic African sanctuary environment was also negatively affected; the orphans were unable to sustain play and more frequently became aggressive as compared to mother-reared juveniles [18].

Socio-emotional skills encompass a wide range of competencies related to how individuals build and maintain relationships, function socially within a group, and understand and regulate emotions [19]. Of such skills, empathy represents a key socio-emotional ability that appears to be compromised by maternal loss in humans [8] and non-human primates [20]. We use the term empathy here to broadly refer to the capacity to share, understand, or respond with concern to the emotional state of others [21,22]. Consolation—the offering of friendly contact by a third party to a distressed individual (such as after a fight) is considered a reliable behavioural marker of empathy [23]. Crucially, consolation has a stress-reducing function for the recipient [22] without any obvious benefits to the actor, supportive of its prosocial function. Among primates, the stress-alleviating effect of being consoled has been demonstrated in several species, including bonobos [24–27], chimpanzees [28] and Tonkean macaques (*Macaca tonkeana*) [29]. Research conducted with bonobos housed at the facility where the present study took place revealed that compared to mother-reared peers, orphan juveniles show lower consolation tendencies and reduced socio-emotional competence, as measured by the number of close affiliative partners, tendency to initiate and sustain play behaviour, and ability to cope with emotional distress [24,25]. Evidence that immature apes engage in consolation suggests that the behaviour may not depend on sophisticated cognitive mechanisms, and there are broader, ongoing debates about levels of cognitive complexity involved in empathetic responses [22,30]. However, as consolation requires the ability to attend to others’ states, it can indicate investment in social relationships and thus is used here as a proxy for social functioning.

Despite the broadly detrimental effects of early maternal loss, opportunities for peer interaction may provide social buffering to support the development of social skills. A recent study of wild mountain gorillas (*Gorilla beringei beringei*) found that, against predictions, orphans did not face pronounced social adversity or fitness costs following maternal loss. Rather, a subsequent increase in their social integration and relationship strength suggested they were able to cope with maternal loss through social group buffering [13]. Morrison and colleagues also found that while early life adversity (including parental loss) predicts high mortality in early life, it had no effect on the adult longevity of mountain gorillas [31]. In captive settings, rehabilitation from the early life trauma of maternal loss is possible through surrogacy and nursery rearing practices. For instance, research with captive chimpanzees revealed that behavioural and social deficits of orphans (who required nursery-rearing to survive) might be partially mitigated by modern nursery-rearing practices that include receiving surrogate care by human carers along with experiencing social interactions within a stimulating peer-group environment, where they are offered the chance to engage in physical activity in more complex outdoor enclosures (reviewed in [32]). Such rehabilitative environments may even prevent changes in neural structures that can otherwise occur following adverse early rearing experiences [33].

Lola ya Bonobo is an African primate centre, accredited to the Pan African Sanctuary Alliance (PASA) [34] that rescues and rehabilitates orphaned bonobos, victims of the illegal bushmeat and pet trade. It provides a naturalistic environment including large, forested enclosures and social groups that favour the adoption of species-typical behaviour, and follows high-standard sanctuary requirements (e.g. housing, welfare, management and others) as a member of PASA [34]. We stress that we henceforth refer to a ‘sanctuary’ in compliance with the aforementioned criteria, as one cannot generalize the conditions, procedures, facilities, aims and circumstances of all great ape centres in Africa, but also in European and American locations which may self-identify as sanctuaries.

African great ape sanctuaries like Lola ya Bonobo offer a unique opportunity to investigate the effects of social rearing in more ecologically valid environments than can be afforded in captivity. Importantly, such settings combine early surrogate human care of orphaned infants with extensive nutritional and veterinary support, followed by supported integration into socially and environmentally enriching groups. This nurturing setting may thus facilitate rehabilitation processes including improved social functioning over time. Compared to other captive settings, such as zoos, home-range sanctuaries are typically more similar to the wild environment and climate and enable apes to live in large social groups and express species-typical behaviours including foraging and nest-building in large forested enclosures [35–37]. Such potential rehabilitation in sanctuaries of this kind has recently been suggested for chimpanzees, where orphans that experienced early life trauma did not vary in their later-life social integration from their mother-reared counterparts born in the sanctuary [38]. A study on sanctuary-living bonobos and chimpanzees also found that orphans from juvenility to adulthood performed equally well on various cognitive tasks as mother-reared peers and did not show abnormalities in behaviour or stress levels [37]. An example of a European primate rescue centre that follows high standards and is a member of European Alliance of Rescue Centres and Sanctuaries (EARS) is a study on ex-pet and performance chimpanzees that suggests that rehabilitation is possible over time, indicated by increasing social competence and welfare and decreasing abnormal behaviours [39].

Thus far, most research on ape social and/or emotional functioning in similar settings has been cross-sectional (but see [39]). Although this provides relevant snapshots in time, it fails to account for potential developmental trajectories over time; moreover, single timepoints might not be representative of later life stages or outcomes. To address this limitation, the present study examined the socio-emotional development of sanctuary-living bonobos using a longitudinal approach. Building upon and extending existing cross-sectional findings revealing rearing effects from this population [24,25], behavioural observations were compiled across five independent periods of data collection spanning a decade. Analyses explored how rearing, sex and age interact to predict three behavioural markers of socio-emotional functioning: affiliation tendencies, consolation tendencies and the likelihood of being a victim or aggressor in social conflicts. As this sanctuary comprises both wild-born orphans (whose rehabilitation process started at a young age) and mother-reared individuals (who were born at the sanctuary), this setting provides a unique opportunity to investigate longitudinally the impact of rearing on socio-emotional development in an environmentally controlled context. Furthermore, the semi-wild housing provides a more valid social and ecological environment than can be afforded in captivity while still maintaining good degrees of observation visibility and large sample size, thus making it a good compromise between wild and captive environments, as these kinds of observations are challenging to achieve in the wild.

Despite providing unique and precious insights into hominid life histories, longitudinal studies of great ape social development are rare. By taking a longitudinal approach, this study aimed to enhance understanding of factors shaping primate socio-emotional development, and to examine the outcomes of sanctuary rehabilitation efforts for endangered species, including bonobos. Lastly, for conservation efforts the majority of the study site’s bonobos may be released and some already have been released into the wild; therefore, we also aimed to expand the knowledge about their ability to perform vital healthy social functioning.

*Affiliative tendencies*: Given that bonobos are a male-philopatric species in which males depend on sustained maternal support for fitness outcomes [5], we predicted that orphaned male bonobos would exhibit more persistent social deficiencies than their female counterparts or mother-reared peers. Furthermore, wild female bonobos migrate at adolescence and integrate into a new group, facilitated by investing in female–female relationships with recently migrated and older resident females of high- ranking positions [6,40–42], leading to our prediction that female bonobos would be less affected by a lack of maternal support with age. Males show less intra-sexual bonding and instead typically build coalitions with their mothers and other females to receive support in acquiring and maintaining high rank positions [5,6]. Thus, we predicted that with age, males would exhibit decreased affiliative tendencies, whereas females would exhibit increased affiliative tendencies. Finally, we predicted that affiliative tendencies in orphaned compared to mother-reared bonobos would persist at a lower level across age, based on evidence that chimpanzee social phenotypes are relatively stable across the life-span [43]. Alternatively, if the sanctuary provides a rehabilitative setting for improved social functioning (e.g. [37,38]), we would expect an increase in affiliation across age within the orphans particularly.

*Consolation tendencies*: Previous research, including in chimpanzees [44] and in bonobos in this study population [24,25], found that infants and juveniles console more often than their older counterparts. Based on these findings, we anticipated a similar overall developmental decline in consolation behaviour. Chimpanzee consolation tendencies were also found to be relatively stable across the lifespan [44]. We, therefore, investigated whether the lower consolation tendencies observed in juvenile orphans (compared to mother-reared bonobos) in this study population [24,25] would persist with age, or if the orphans’ empathic tendencies would improve over time. Again, based on species-typical patterns, we would expect female orphans to increasingly align with mother-reared bonobos with age, whereas male orphans would exhibit a consistent disparity with their mother-reared counterparts into adulthood. Research on sex differences in consolation remains limited and inconsistent, with conflicting results reported in great apes [24,44–46]. We, therefore, did not have a strong directional prediction regarding sex differences.

*Social conflict risk*: Last, we investigated the likelihood of being a victim or aggressor in social conflicts. Again, based on the species’ natural social ecology, we predicted that older females—the high-ranking core of the group [47]—would be the most likely aggressors. Furthermore, we expected that aggression would be primarily directed at young bonobos, especially male orphans, who are at a disadvantage when trying to integrate into a social group in the absence of maternal support.

## 2. Methods

### 2.1. Study site and subjects

Observations were conducted at Lola ya Bonobo sanctuary in Kinshasa, Democratic Republic of the Congo. Groups at Lola ya Bonobo consist primarily of orphaned victims of the illegal bushmeat and pet trade. Most wild-caught orphans have experienced some form of trauma, poor health, and malnourishment owing to maltreatment prior to their rescue at an estimated age of minimum 2−3 years. Age at the rescue is estimated by dental and weight estimates by the veterinarians of the sanctuary [37]. Along with orphans, groups include some bonobos who were born and mother-reared at the study site, where limited reproduction is allowed to promote naturalistic social settings of mixed-age social groups (following strict guidelines for primate release programmes e.g. by IUCN, PASA).

Alongside rescuing and housing orphaned bonobos at Lola ya Bonobo, Amis des Bonobos du Congo (ABC) manages a reintroduction site called Ekolo ya Bonobo, where fully rehabilitated individuals are released into a protected wild habitat. For eligibility for reintroduction, individuals pass through a series of stages upon rescue (see the electronic supplementary material, box 1). The study site at Lola ya Bonobo features three separate outdoor enclosures that provide a semi-wild environment composed of secondary rainforest, grasslands and swampy areas with ad libitum access to water (by means of a lake, floating stream or a pool). Observations were conducted on all individuals from the three social groups (henceforth G1, G2, G3); these include orphaned as well as mother-reared individuals of all age and sex classes across different time points (G3 was observed in 2016 only). As observational data were collected across multiple years (see below), there are fluctuations in observed individuals (across a total of *n* = 83) and group compositions based on births, deaths, reintroductions into the wild and transfer between groups following veterinary and management decisions (electronic supplementary material, table S1).

### 2.2. Data collection and processing

Data were collected over a decade during five time points in the years 2011, 2012, 2016, 2019 and 2021, respectively. Each time point comprised 2−3 month observation periods (electronic supplementary material, table S2). Observations were led by Z.C. (2011, 2012) and S.K. (2016, 2019, 2021) with assistance of Pitshou Nsele Kayanga (2011, 2012) and Heritier Izansone (2019, 2021). Across all observation periods, consistent methods of data collection and ethograms were applied (see below for a deviation regarding affiliation scans in 2019). Observations included social affiliation scan sampling, all-occurrences of post-conflict/post-distress (hereafter PC/PD) focal video sampling and all-occurrences of agonistic conflicts followed by an identifiable victim response. PC/PD recordings were prioritized over scan sampling if they occurred simultaneously. The identities of the aggressor and victim as well as the type of aggression were recorded on a dyadic level per group and observation period (see the electronic supplementary material for ethogram and definitions). Observations of social affiliations only began with the presence of at least one-quarter of the group members to allow for sufficient opportunities to engage in social interactions to estimate socializing. All instances of aggressions were recorded when at least the aggressor and victim were visible.

#### 2.2.1. Affiliation scans

Instantaneous scan samples of all visible social affiliations at the group level were recorded throughout the day, outside of feeding times (when PC/PD recordings were the primary focus, and to avoid overestimation of proximities influenced by food location). The presence of each individual as well as dyadic proximities and interactions of state behaviours were recorded at each scan point (i.e. contact sit, within arm’s reach, play, groom, sexual contact; see the electronic supplementary material, table S6 for an ethogram). Intervals between scan points remained consistent within each observation period, but differed between some periods, that is, affiliation scans were conducted every 10 min in observation periods 2011 and 2012, and every 20 min in 2016 and 2021. The data collection in 2019 deviated modestly from other years; however, the resulting raw data remained constant. In 2019, social affiliation was recorded using a 10 min focal scan [48], consisting of ten 1 min scan points whereby affiliative behaviours (equal to all observation periods) the focal was involved in were recorded. A mean dyadic affiliation score was calculated per individual and observation period and controlled for overall social tendencies per group and observation period, that is, (*N* scan points a dyad interacted/*N* scan points a dyad was present) divided by the group mean across individual dyadic affiliation scores per observation period (adapted from [24,49]). As all behaviours required body contact or proximity of one arm’s length, they were all combined into the affiliation score, representing dyadic proximities. Across all study periods, we collected a total of *n* = 6337 social affiliation scans (see the electronic supplementary material, table S7).

#### 2.2.2. Post-conflict and post-distress focals

To measure consolation, PC and PD focal observations were collected on victims of aggression with high-definition hand-held camcorders. As conflicts occurred spontaneously, we conducted all occurrence-focal sampling [48] on individuals of all age classes. The victim was identified as the recipient of aggression resulting in clear victim response behaviours such as a high-pitched distress scream (see the electronic supplementary material, table S3). In case of a spontaneous tantrum, or when the conflict occurred out of sight, the focal recording was marked as a PD observation. Previous observations at this study site demonstrated that consolation behaviour mostly occurred within the first 5 min of a PC/PD (70.7% within the first 2 min, 84.6% within the first 5 min; [24]), indicating that 5 min PC/PD focal follows were sufficiently long. Henceforth, a PC period was defined as a period of 5 min after the onset of a conflict between two opponents leading to a clear losing party of the conflict (i.e. the victim, see the electronic supplementary material, S1.2). A PD period did not include a conflict but started with a spontaneous outburst of distress and was observed for 5 min as well. During behavioural coding (electronic supplementary material, S1.2.1), consolation was defined as the first spontaneous affiliative body contact offered by a third party bystander to the victim of a conflict or spontaneous distress during the PC/PD period [25,50]. A detailed ethogram was established to code for affiliative behaviours and aggressive behaviours of different intensities (electronic supplementary material, tables S4 and S5). Subsequently, affiliative behaviours were identified as consolation behaviours (meeting criteria of third party initiated affiliative bodily contact). Previous studies, including the 2011 observation period of the present study [24] have demonstrated the presence of consolation by means of the post-conflict-matched control method (e.g. [24,50]), we thus here renounced the anew use of matched-control recordings.

#### 2.2.3. All-occurrence aggression

Additionally, we manually recorded all observable aggressions (including aggressions occurring during PCs) using the same ethogram as for PC recordings (electronic supplementary material, table S4) to identify aggression and victim rates throughout three observation periods (2016, 2019, 2021).

### 2.3. Data analysis

Analyses were conducted with generalized linear mixed models (GLMMs) in RStudio (R version 4.3.1 [51]; open access to our data and code [52]). We used the function glmmTMB of the equally named package (version 1.1.7; [53]) for models 1 and 3. The function glmer of the package lme4 (version 1.1.30; [54]) was used for model 2. Datasets of all models comprised observations across several observation periods (but see the electronic supplementary material, table S2), whereby all individuals were present during at least one. As we had repeated observations of some individuals, subject ID and group per observation period were included as random intercepts in all models.

Consistent with the hypothesis that empathic tendencies such as consolation are socially biased [55] previous studies including from bonobo groups housed at the site of the present study have revealed that social closeness and kinship predict consolation tendencies in bonobos and chimpanzees [24,56]. Orphans do not have the same opportunities to engage in such close social relationships as they would with their kin. Consequently, all interactions between matrilinear kin (mother-offspring, maternal siblings) were removed from the analysis in order to facilitate comparisons between the two rearing categories (orphan and mother-reared). Paternities were not considered as they were only known for some of the sanctuary-born individuals. The two rearing categories differed in their age range (orphans: 3−28 years; mother-reared 3−16 years). Therefore, to ensure comparability, we only included individuals up to the maximum age of mother-reared subjects in the dataset, respectively, that is, aged up to 16 years for model 1 (affiliation tendency) and model 3 (victim tendency) and up to 14 years for model 2 (consolation tendency).

Model stabilities were assessed using a function provided by Roger Mundry (personal communication, 2022), which involved dropping one random effect at a time from the data. The resulting estimates from these subsets were then compared to the estimates obtained from models using the full dataset. *Minimum* and *maximum* values for each fixed and random effect estimate are presented in all results tables. These values show the full range of estimates obtained from the model comparisons. A relatively narrow range either above or below 0 would indicate stable significant effects. Covariate predictors were *z*-transformed to increase the likelihood of model convergence. Factor variables were dummy- coded and centred prior to their inclusion as random slopes. The R function drop1 with argument ‘test’ set to ‘Chi^2^’ was used to test the effects of the predictor in all models. To test the overall effect of main predictors and to avoid ‘cryptic multiple testing’ [57], each full model was compared to an otherwise identical null model that lacked the main predictors in the fixed effects. Confidence intervals of model estimates were obtained using 1000 parametric bootstraps (function simulate of the packages glmmTMB and lme4). Collinearity was checked based on the models lacking the interaction using variance inflation factors (VIF) with the R package ‘car’ [58].

Inter-observer and inter-coder reliability were both assessed using Cohen’s κ coefficient [59]. For consolation coding, a subset of 10−15% of videos across observation periods was coded by an additional independent coder trained on the ethogram to identify all social behaviours as well as the initiating party to identify the occurrence of consolation (i.e. third party initiated affiliative contact behaviours). For the identification of consolation, results indicated excellent agreement (consolation occurrence: κ>0.84; partner identity: κ>0.99). For social affiliation, observers conducted live identical scan samples in the field. For partner identity and social behaviours, results indicated excellent agreement (partner identity: κ>0.81; social behaviour: κ>0.86).

#### 2.3.1. Model 1: affiliation tendency

To estimate the effects of sex, age and rearing on affiliative tendency across all observation periods, we fitted a GLMM with a beta error distribution and logit-link function [60,61], which allows for the analysis of proportional data.

To test for interactions across multiple two-way combinations of our fixed effects, we included all two-way interactions between sex, age, and rearing (sex*age+rearing*age+sex*rearing), as well as all respective main effects. A full three-way interaction was not included owing to limited interpretability of such a complex parameter on a relatively small dataset. The response variable for model 1 was individual mean affiliation scores (see the electronic supplementary material, table S8 for a model overview). The initial model 1 contained all theoretically identifiable random slopes; however, to facilitate convergence and lessen the complexity of the model, we removed correlations between random intercepts and slopes. The model was not overdispersed (dispersion parameter: 0.652). Checks for collinearity revealed no collinearity issues (maximum VIF = 1.155). Stability checks revealed that all estimates for this model were relatively stable. The sample size for model 1 comprised 160 observations, across *n* = 74 individuals (female *n* = 35, male *n* = 39, orphan *n* = 55, mother-reared *n* = 19).

#### 2.3.2. Model 2: consolation tendency

To estimate the effects of sex, age and rearing on consolation tendency across all observation periods, we analysed data on a dyadic level using a GLMM with a binomial distribution [62]. Each observation row represented a bystander-victim combination per group and year, where a bystander had at least one opportunity to console a given victim. We excluded *n* = 6 juveniles for whom we lacked affiliation scan data (observation period 2012: LY, MAY, MAK; observation period 2019: BAR, KIT, LIK) from the analysis. The response variable for model 2 comprised a two-columns matrix using the R function ‘cbind’ representing the number of consolations and non-consolations per bystander-victim combination, which was thus analysed as a binomial distribution. The full model included individual characteristics of the bystander and victim (sex, age, rearing), respectively. To control for the bystander-victim relationship, we included dyadic affiliation scores calculated from social affiliation scans per dyad, group, and observation period as fixed effects. As in model 1, we proceeded with three two-way interactions as opposed to one three-way interaction to avoid overcomplexity and limited interpretability (see the electronic supplementary material, table S9 for an overview of the full model). All theoretically identifiable random slopes were included in the original model 2, however, to enable model convergence and reduce its complexity we subsequently removed the correlations among random intercepts and slopes. After the model was fitted, a Q-Q plot (quantile-quantile plot) [63] of residuals and residuals plotted against fitted values was visually inspected to check if the assumptions of normally distributed and homogeneous residuals were met [64]. These indicated no deviations from these assumptions. No collinearity issues were found in collinearity checks (maximum VIF = 1.364). Stability checks revealed the model had acceptable stability. Based on a total of *n* = 1071 PC and PD focal follows, the model consisted of *n* = 1590 dyadic victim-bystander observations across *n* = 61 individuals (female *n* = 30, male *n* = 31, orphan *n* = 45, mother-reared *n* = 16).

#### 2.3.3. Model 3: victim likelihood

To investigate which factors may predict the likelihood of being aggressed across years, we conducted additional analyses based on dyadic aggressions per group and year using a GLMM [62] with beta error distribution and logit-link function [60,61]. The response variable was victimhood proportion, calculated as the count of being a victim within a victim-aggressor dyad and respective group and observation year, divided by the total counts of observed aggressions within the respective group and observation year. Prior to fitting the model, we transformed the response closer to 0.5 to avoid values being exactly zero or one [65].

As orphaned males were expected to be likelier victims of aggression and older females were expected to be more likely aggressors, we included the interactions of victim-sex * victim-rearing and aggressor-sex * aggressor-age as fixed effects. Further fixed effects were victim-age and aggressor-rearing (see the electronic supplementary material, table S10 for an overview of model 3). While several random slope components were theoretically identifiable, our data frame was too limited in size to allow for their reliable inclusion, which further led to convergence issues. To allow for model convergence and limit model overcomplexity and instability leading to unreliable estimates with greater uncertainty, we proceeded with a parsimonious modelling approach and did not include a more complex random effects structure. Random slopes of the aggressor as well as victim rearing and sex within group-year did not affect model convergence and remained in the model (excluding correlations among random intercepts and slopes). The model was overdispersed (dispersion parameter: 2.225), thus results are to be interpreted with caution. Checks for collinearity revealed no collinearity issues (maximum VIF = 1.187). Stability checks revealed that all estimates for model 3 were relatively stable. A total of *n* = 1042 aggressions were observed across *n* = 3 different observation periods (2016, 2019, 2021). The model included *n* = 1853 observations with *n* = 76 aggressors and *n* = 62 victims (whereby individuals can be represented in both categories).

## 3. Results

### 3.1. Model 1: affiliation tendency

**Figure 1.**
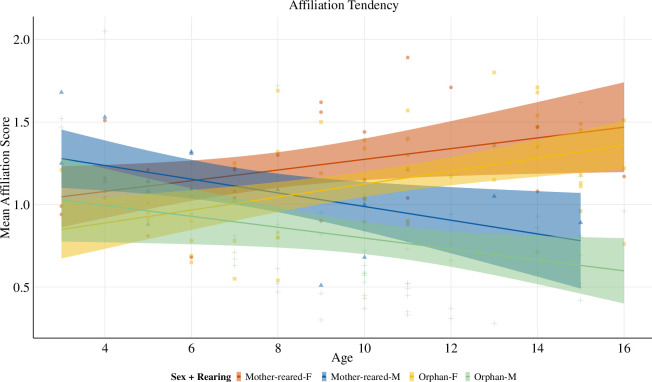
Scatterplot of significant main effects of model 1: investigating effects of rearing and the interaction between sex and age on affiliative tendencies. Raw data of individual mean dyadic affiliation scores controlled for group and year across age sex and rearing. Females increased their social affiliation with age, while males decreased social affiliation with age. Mother-reared showed higher affiliation scores than orphans. *x*-axis: age in years; *y*-axis: data points present individuals’ mean dyadic affiliation scores, including observations of all individuals (*n* = 74) across the five different observation periods, only some of which are represented with repeated observations across periods, owing to demographic changes. Colours correspond to sex and rearing as indicated in the legend (F = female, M = male). Points correspond to respective individual mean dyadic affiliation scores.

Overall, model 1 provided a significantly better fit than a null model lacking the predictors (full-null model comparison: $\chi^{2}$ = 43.78, *p* < 0.001; see the electronic supplementary material, table S11 for full model results including non-significant interactions). We further proceeded with a reduced model lacking the non-significant interactions (rearing*sex, rearing*age) to investigate the main effects of rearing (full-reduced model comparison: $\chi^{2}$ = 4.150, *p* = 0.126). More specifically, we found a significant interaction between sex and age on social affiliation (estimate ± s.e. = −0.265 ± 0.056, *z* = −4.735, *p* < 0.001), with females showing increased dyadic affiliation tendencies with age, and males showing decreased tendencies with age (figure 1; table 1). Furthermore, orphans showed lower affiliation tendencies than mother-reared individuals (estimate ± s.e. = −0.204 ± 0.087, *z* = −2.342, *p* = 0.022; see the electronic supplementary material, figure S2 for a model output plot).

**Table 1.** Results of model 1: reduced GLMM for affiliation tendency (estimates and standard errors, together with confidence intervals (CIs), results of likelihood ratio tests, and the range of estimates obtained when dropping levels of random effects one at a time). (Significant *p*-values < 0.05 are shown in bold.)

term	estimate	s.e.	lower CI	upper CI	*z*	d.f.	*p*	min	max
(intercept)	−2.818	0.105	−3.018	−2.626	−26.805	^a^	^a^	−2.865	−2.753
sex^b^	−0.346	0.078	−0.495	−0.185	−4.426	^a^	^a^	−0.385	−0.306
age^c^	0.128	0.041	0.048	0.209	3.125	^a^	^a^	0.112	0.163
rearing^d^	−0.204	0.087	−0.369	−0.036	−2.342	1	**0.022**	−0.322	−0.158
sex^b^: age^c^	−0.265	0.056	−0.368	−0.162	−4.735	1	**<0.001**	−0.323	−0.216

^a^ Not indicated owing to limited interpretability.

^b^ Male (sex dummy coded and centred with female as the reference level).

^c^ *z*-transformed to a mean of 0 and a standard deviation of 1.

^d^ Orphan (rearing dummy coded and centred with mother-reared as the reference level).

**Figure 2.**
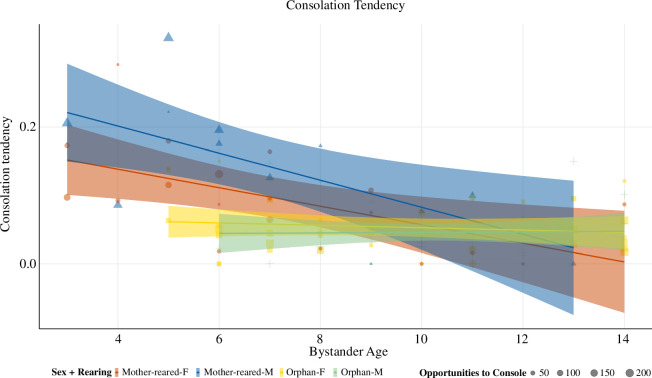
Scatterplot of significant main effects of model 2: investigating effects of the interaction between bystanders’ age and rearing on consolation tendency. Raw data of individual mean consolation tendency per group and observation period. Mother-reared showed a developmental decline of consolation tendencies, while orphans had consistent, but lower consolation tendencies across age (within the range of older mother-reared). *x*-axis: age in years; *y*-axis: bystander mean consolation tendency per group and observation period. Data points present individual consolation tendencies of all individuals included in the model (*n* = 61) across the four different observation periods, only some of which are represented with repeated observations across periods, owing to demographic changes. Colours correspond to sex and rearing as indicated in the legend (F = female, M = male).

**Table 2.** Results of model 2: full GLMM for consolation tendency (estimates and standard errors, together with confidence intervals, results of likelihood ratio tests, and the range of estimates obtained when dropping levels of random effects one at a time). (Significant *p*-values < 0.05 are shown in bold.)

term	estimate	s.e.	lower CI	upper CI	*z*	*χ* ^2^	*p*	min	max
(intercept)	−3.128	0.345	−4.083	−2.688	−9.053		^a^	−3.587	−2.858
bystander age^c^	−0.595	0.149	−0.945	−0.366	−3.982		^a^	−0.768	−0.442
bystander sex^b^	0.449	0.352	−0.04	1.318	1.273		^a^	0.158	0.811
bystander rearing^d^	−0.354	0.317	−0.759	0.499	−1.118		^a^	−0.634	0.077
victim age^c^	−0.18	0.141	−0.327	0.036	−1.277	2.6	0.107	−0.268	−0.078
victim sex^b^	−0.056	0.142	−0.248	0.325	−0.395	0.083	0.774	−0.114	0.026
victim rearing^d^	−0.004	0.216	−0.331	0.454	−0.02	0.113	0.736	−0.15	0.245
dyadic affiliation^c^	0.355	0.059	0.264	0.432	5.995	64.006	**<0.001**	0.298	0.387
bystander age^c^ : bystander sex^b^	−0.074	0.148	−0.412	0.231	−0.498	0.379	0.538	−0.181	0.2
bystander age^c^ : bystander rearing^d^	0.597	0.16	0.355	1.006	3.725	18.362	**<0.001**	0.418	0.743
bystander sex^b^ : bystander rearing^d^	−0.282	0.41	−1.272	0.407	−0.687	1.038	0.308	−0.67	0.032

^a^ Not indicated owing to limited interpretability.

^b^ Male (sex dummy coded and centred with female as the reference level).

^c^ *z*-transformed to a mean of 0 and a standard deviation of 1.

^d^ Orphan (rearing dummy coded and centred with mother-reared as the reference level).

### 3.2. Model 2: consolation tendency

The full model was significantly better at predicting consolation tendencies, when compared to a null model lacking its predictors (full-null model comparison: $\chi^{2}$= 30.828, *p* < 0.001). Although the full model resulted in non-significant interactions of sex*rearing and sex*age, we remained with the full model as it fitted significantly better than the reduced model with these interactions removed (full-reduced model comparison: $\chi^{2}$= 37.794, *p* = 0.003). Model estimates revealed that the interaction between bystander age and bystander rearing significantly predicted the tendency to console (estimate ± s.e.= 0.597 ± 0.16, *z* = 3.725, *p* < 0.001). As shown in figure 2 and the plotted model output (see the electronic supplementary material), mother-reared bonobos showed a decrease in consolation tendency with age while orphaned bonobos showed more consistent but lower consolation tendencies across age (only overlapping with older mother-reared bonobos). Although not under investigation, the control variable of bystander-victim dyadic affiliation tendency positively predicted consolation (estimate ± s.e. = 0.355 ± 0.059, *z* = 5.995, *p* < 0.001). See table 2 for an overview of model 2 results.

### 3.3. Model 3: victim likelihood

Overall, the model of victim aggression tendency was significantly better at fitting the data as compared to a null model lacking main predictors (full-null model comparison: $\chi^{2}$= 65.488, *p* < 0.001). Results refer to a reduced model (lacking the non-significant interaction between victim rearing and victim sex) to examine the main effects (reduced-full model comparison: $\chi^{2}$= 0.481, *p* = 0.488).

The interaction of aggressor age and aggressor sex significantly predicted victim likelihood, that is, victims are more likely to be aggressed by older females (estimate ± s.e. = −0.240 ± 0.049, *z* = −4.886, *p* < 0.001). Mother-reared bonobos showed higher aggression than orphans (estimate ± s.e. = −0.173 ± 0.066, *z* = −2.635, *p* = 0.011). Regarding victims, young bonobos (estimate ± s.e. = −0.085 ± 0.025, *z* = −3.339, *p* = 0.007) and males (estimate ± s.e. = 0.124 ± 0.045, *z* = 2.747, *p* = 0.006) were aggressed most as opposed to older individuals and females (table 3; see the electronic supplementary material for a model output plot).

**Table 3.** Results of model 3: reduced GLMM of the likelihood of being a victim of aggression (estimates and standard errors, together with confidence intervals, results of likelihood ratio tests, and the range of estimates obtained when dropping levels of random effects one at a time). (Significant *p-*values < 0.05 are shown in bold.)

term	estimate	s.e.	lower CI	upper CI	*z*	d.f.	*p*	min	max
(Intercept)	−5.563	0.077	−5.713	−5.410	−72.513	^a^a	^a^a	−5.609	−5.471
victim age^c^	−0.085	0.025	−0.133	−0.035	−3.339	1	**0.007**	−0.113	−0.071
victim rearing^d^	0.123	0.067	−0.005	0.249	1.818	1	0.079	0.082	0.155
victim sex^b^	0.124	0.045	0.036	0.212	2.747	1	**0.006**	0.087	0.158
aggressor age^c^	0.278	0.037	0.199	0.356	7.573	^a^a	a	0.259	0.319
aggressor sex^b^	−0.187	0.051	−0.290	−0.093	−3.652	^a^a	a	−0.238	−0.137
aggressor rearing^d^	−0.173	0.066	−0.311	−0.043	−2.635	1	**0.011**	−0.213	−0.138
aggressor age^c^ : aggressor sex^b^	−0.240	0.049	−0.335	−0.141	−4.886	1	**<0.001**	−0.297	−0.209

^a^ Not indicated due to limited interpretability.

^b^ Male (sex dummy coded and centered with female as the reference level).

^c^ z-transformed to a mean of 0 and a standard deviation of 1.

^d^ Orphan (rearing dummy coded and centered with mother-reared as the reference level).

## 4. Discussion

Using longitudinal data collected across a decade of observations, we investigated the effects of rearing environment (specifically, early maternal loss) as well as sex and age, on the socio-emotional functioning of sanctuary-living bonobos from juvenility to adulthood. We found that, overall, orphan bonobos have lower socio-emotional tendencies than mother-reared bonobos (however, within their range). A developmental decline in consolation was found in mother-reared bonobos, while orphans had consistently lower consolation tendencies across age. Affiliation tendencies diverged between the sexes with age, and older females and mother-reared bonobos were the most aggressive. Young bonobos were at a higher risk of aggression, but contrary to expectations, victims' rearing background did not predict their aggression risk.

Our finding that females increased while males decreased their social affiliation with age is consistent with bonobo social ecology—whereby unrelated females, who typically migrate at adolescence, build strong coalitions at the core of the group [40]. The developmental decline in male affiliative tendencies likewise corresponds to species-typical patterns, whereby adult males tend to stay peripheral to the group and, other than strong bonds with their mothers, show less pronounced bonding with group members with age. To enable comparisons of the two rearing backgrounds; however, we did not include kin-relationships; thus, the effect of mother-son bonds is not represented in the results. Future research is needed to test the extent to which changes in social affiliation with age reflect shifts in how selective (or not) individuals become towards certain social partners (such as mothers or other adult females).

With respect to rearing, we predicted that the effects of rearing would be less pronounced for females given their enhanced social status in bonobos and that similar to patterns observed in wild migrating females, their social integration would increase with age [6,40–42]. By comparison, we expected that orphaned males, who lack maternal support, which has been shown to be crucial for their social integration [5], should continue to exhibit social deficiencies throughout their lives. Despite this, our results did not reveal interactions between rearing with either sex nor age, respectively. It is unclear why this is the case; however, we did find that overall, mother-reared bonobos had higher affiliation tendencies than orphans, as predicted. This is consistent with other evidence from rescued pet and entertainment chimpanzees that early maternal loss has long-lasting effects on social functioning, indicated by reduced affiliative behaviour and grooming activity [16,17], or non-sustained play behaviour in orphaned chimpanzees [18] housed in a sanctuary comparable to the present study’s setting. Although same-species comparisons would be preferable, the most relevant literature is available for chimpanzees. Despite the phylogenetic proximity of the two species, we are aware that direct comparisons here and henceforth need to be interpreted cautiously given some of the differences in their behavioural ecology.

We further analysed tendencies to console others in distress, considered a behavioural marker of empathy and a more general indicator of socio-emotional functioning [20]. Although the underlying mechanisms remain challenging to identify, evidence that consolation is targeted towards distressed parties and effectively alleviates their distress suggests that it involves the capacity to not only recognize but appropriately respond to other’s needs (as well as a capacity to distinguish self from other, to avoid becoming personally distressed in the face of another’s situation) [20]. We found an effect of rearing, whereby orphans showed consistently lower consolation tendencies across time than mother-reared. Interestingly, rearing also seemed to disrupt expected developmental trajectories of consolation: only in mother-reared bonobos, but not orphans, did we see the expected developmental decline in consolation over time that has been previously observed in captive apes (bonobos: [24]; chimpanzees: [44]; western gorillas: [45]). Importantly, this also meant that overall, orphan consolation tendencies remained within the range of mother-reared bonobos, even if they were initially lower during immaturity.

Although the present observational study cannot discern underlying mechanisms, the apparent developmental pattern of higher consolation tendencies during immaturity may reflect an intrinsic drive of young apes to attend to and orient towards others’ emotional states and situations. While consolation functions to relieve tension or distress in the recipient (reviewed in [66]), in this respect it may also confer benefits to actors, such as opportunities for immatures to learn about their social worlds and relevant social events. If affiliating with victims enhances the affiliative relationship between the victim and consoler, it may also enable immatures to expand their social networks. In chimpanzees, males initially show a social bonding strategy to affiliate with many partners, which then shifts with age to invest in fewer but stronger relationships [67]. Although this remains to be tested, it is possible that consolation efforts, too, start off initially broad but become selectively geared towards close social partners with age. Further research, examining the affiliative consequences of consolation and its selectivity with age is needed to test this.

Our results highlight that early life stress may impact species-typical developmental trajectories. Nevertheless, although orphans showed lower levels of consolation than mother-reared peers, their tendencies to console were not altogether absent, suggesting that orphans are not ambivalent to others’ states, and are sometimes able to offer appropriate prosocial responses. The fact that their consolation tendency moreover lay within the range of mother-reared, albeit at the lower end, suggests they may have sufficient skills to cope within their social groups.

A key question is whether orphans have impaired socio-emotional functioning as compared to mother-reared or are inhibited from expressing it. Young orphans, who lack the support of their mothers and have yet to establish social bonds, may behave differently than young mother-reared bonobos who may learn that they can rely on their mother’s support. Thus, avoiding socially risky situations like post-conflict periods may also be a coping strategy for traumatized young orphans lacking social support, akin to conflict-avoidance (i.e. reducing social interactions) and tension-reduction (i.e. reducing aggressive behaviours and increasing affiliative behaviours) observed in chimpanzees adapting to limited space [68]. Bonobos may, too, exhibit this flexibility in certain social situations. Nevertheless, this would not clearly explain why older orphans continued to show low levels of consolation when presumably their social relationships were more established.

Regarding sex differences in consolation tendency, our results were consistent with some previous studies that found no sex differences (bonobos: [24]; chimpanzees: [44]). Nevertheless, as noted, ape studies do vary in regard to sex differences, with some finding no effects, while others finding a female and/or dominant male bias [45,46]. This suggests that potential sex variation in consolation might be contextually dependent on a range of demographic factors, including social composition, group tolerance and social-dominance relationships, which may vary within and between groups as well as across species. Further work is needed to address this.

Finally, we also examined longitudinal trajectories of receiving as well as committing aggression, another relevant aspect of social functioning. In line with our predictions, males and young bonobos were more likely to be victims of aggression, whereas older bonobos and females were most frequently aggressors relative to young ones and males. These patterns appear to reflect the species-typical social structure of high-ranking adult females and the lower status of most males [47] and are in line with personality research on wild bonobos that found higher aggression in older individuals [69]. The higher aggression in mother-reared bonobos may further reflect their higher rank and/or more central social position compared to orphans. In contrast to our prediction, victim-rearing did not significantly affect the likelihood of being aggressed, indicating that orphans are not targeted more than mother-reared despite their seemingly lower social tendencies indicated by our other results. However, a trend hints towards orphans being more likely to be aggressed, which may be explained by their lack of maternal support, especially when younger.

Taken together, our results support the overall hypothesis that although early life adversity impacts socio-emotional functioning in this ape species, orphaned bonobos still show evidence of social functioning, albeit at a reduced level. Prior to their rescue by the sanctuary, most orphans will have probably been forcibly removed from their mothers, who will have probably been killed in their presence along with other group members, as well as subsequently experiencing neglect and potentially also abuse. Yet, despite these traumatic experiences, our study suggests that their social and consolatory tendencies, an aspect of socio-emotional functioning, overlap within the lower range of mother-reared peers. Another study by Wobber & Hare [37] experimentally showed that orphaned bonobos at this same study site were also equivalent in their cognitive and behavioural skills as mother-reared peers. While this is encouraging, it should be noted, however, that their measures, as well as our own, may not have picked up other socio-emotional factors that can be influenced by rearing. Further behavioural and physiological work is needed to pinpoint which aspects of their social functioning are impacted and the mechanisms underlying this.

Most orphans in this study arrived at the sanctuary at around 2–5 years old, thus it is also possible that the initial phase of mother-infant care they received, prior to the moment of capture, may have been a buffer for building long-term resilience. As we know from the human literature, the age and duration of deprivation are critical for informing the severity of trauma experienced [8]. Rehabilitated chimpanzees, who were deprived at an earlier age, showed long-term deficits in social competence, compared to those who stayed with their mothers for longer and subsequently with peers [15]. Arriving at the sanctuary from around 2 years upwards may thus enable greater resilience. There is also evidence that being reared in a social group within a natural environment can improve social deficiencies in apes, as found in wild orphaned mountain gorillas and chimpanzees [13,70]. As suggested for chimpanzees [38,71], the nurturing social environment of Lola ya Bonobo sanctuary, which includes the additional surrogacy, nutritional and veterinary support offered by human carers, may compensate for early-life rearing adversities and facilitate social rehabilitation.

*The rehabilitative function of great ape sanctuaries*: Although our study is unable to make strong inferences regarding great ape rehabilitation, our results do indicate that the orphan bonobos are able to socially function in their groups and show social and consolatory tendencies within mother-reared range, even if to a lower degree. As the only bonobo sanctuary in the world, Lola ya Bonobo plays an essential role in the conservation of this endangered species. Young orphaned apes traumatised by the illegal bushmeat and pet trades [72] are given a second chance in a supportive sanctuary environment (by our sanctuary definition above). Importantly, mother-reared bonobos in our study represent the first generation of offspring, raised by orphan mothers. The fact that their social functioning patterns seem to be in line with the social ecology of their wild counterparts highlights the crucial role that sanctuaries like this play in rehabilitation of apes across generations. Many of these bonobos—including several subjects in the current study—are successfully being reintroduced into the wild, including orphans. Understanding their ability to function in a complex social environment is vital to these efforts. The present study may aid such facilities by providing this information based on systematic investigations.

Although many of these orphans cope relatively well in the social systems, investigating the occurrence of more severe aggressions would shed light on potential difficulties adult orphan males may face without maternal support. Future research should address these issues, including dominance hierarchies as well as social network analysis, personality traits and physiological measures of stress (e.g. cortisol measures) to complement behavioural data and to identify the challenges and strategies orphans encounter in their social integration across the lifespan.

## 5. Conclusion

Through a much-needed longitudinal approach, our study addressed the developmental trajectories of socio-emotional functioning of sanctuary living bonobos across the lifespan. Overall, the results provide key insights into understanding the factors shaping great ape socio-emotional development and the role that sanctuaries like this play in the rehabilitation process. Future research on individual differences in predictors of life outcomes is needed to aid sanctuary management strategies in their conservation efforts of rewilding healthy bonobos. Range country great ape sanctuaries such as this, which align with high standard husbandry and welfare requirements, play a major role in protecting our endangered closest relatives from the ever-rising anthropogenic threat of extinction. With rapidly declining numbers of apes in the wild and increasing numbers of orphans overwhelming range-country primate rehabilitation centres, more research should be directed at assisting these critically required conservation efforts wherever possible.

## Data Availability

Data and code can be accessed via the Figshare Digital Repository. [52]. Supplementary material is available online [73].

## Authors' Notes

Prior to his tragic passing, Frans de Waal both approved and contributed to the initially submitted manuscript, therefore he remains as a co-author of the present paper.

## Dedication

With great sadness and gratitude, we would like to dedicate this paper to our co-author and mentor, the late Frans de Waal, whose ground breaking work, generous support, and mentorship both inspired and enabled this research and has paved the way for generations to follow.
